# Design considerations for representing systems biology information with the Systems Biology Graphical Notation

**DOI:** 10.1515/jib-2022-0024

**Published:** 2022-07-04

**Authors:** Falk Schreiber, Tobias Czauderna

**Affiliations:** Department of Computer and Information Science, University of Konstanz, Konstanz, Germany; Faculty of Information Technology, Monash University, Clayton, Australia; Faculty of Applied Computer Sciences & Biosciences, University of Applied Sciences Mittweida, Mittweida, Germany tobias.czauderna@hs-mittweida.de

**Keywords:** design, SBGN, systems biology

## Abstract

Visual representations are commonly used to explore, analyse, and communicate information and knowledge in systems biology and beyond. Such visualisations not only need to be accurate but should also be aesthetically pleasing and informative. Using the example of the Systems Biology Graphical Notation (SBGN) we will investigate design considerations for graphically presenting information from systems biology, in particular regarding the use of glyphs for types of information, the style of graph layout for network representation, and the concept of bricks for visual network creation.

## 1 Introduction

Design and biology can interact and mutually benefit each other in many ways: from designing and using graphical representations and visualisations of biological information [1, 2] to biomimicry, a type of bio-inspired design [3] to biodesign which aims to incorporate living organisms as components into design [4]. As Michael Gross writes: “Nature inspires art, but conversely, art can also aid biological understanding, which, in turn, can help the appreciation and conservation of art works” [5].

Here we will focus on the first area – design of visualisations – as an enormous amount of information in biology comes in a visual form such as textual sequences, images, diagrams, and networks. Often the graphical representation is not or only weakly predefined but can be changed by a designer or user in order to make the visual presentation not only better understandable but also aesthetically more pleasing.

**Figure 1:**
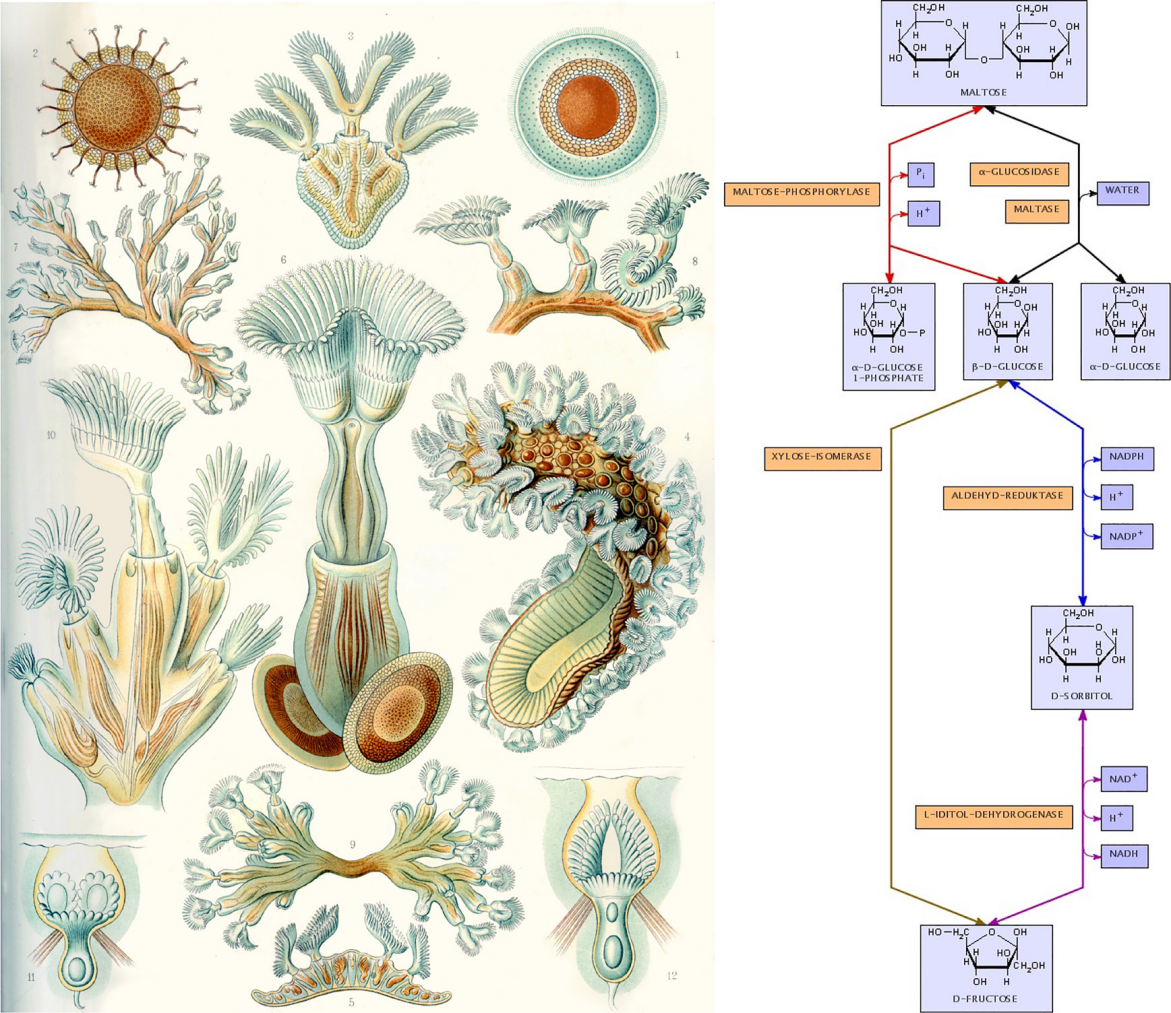
A drawing by Ernst Haeckel (left, Bryozoa minor restoration from [6]) and a figure produced with the BioPath system [7], the electronic version of Gerhard Michal’s pathway poster [8] using an automatic layout algorithm [9] (right).

A broad range of informative and aesthetic examples exists such as the work of Ernst Haeckel, who created hundreds of detailed drawings and watercolours of his scientific findings [6] more than hundred years ago (see also the book about his work by Willmann and Voss [10]) or the work by Gerhard Michal, who created widely used drawings of metabolic pathways [8, 11], his famous poster has been printed over a million times. For examples see also Figure 1.

Design principles have been used to present biological information for a long time, although not always by explicit incorporation of such principles in the visualisation process. The importance of design principles for good (i.e., informative and aesthetically pleasing) visualisations has been discussed and several sets of principles have been proposed. One prominent example is the series of *Points of View* columns on data visualisation published since 2010 in Nature Methods (starting with Bang Wong’s colour coding column [12]) which covers a broad range of topics such as simplification, colour avoidance, typography, labels, layout, data exploration, two and three dimensions, the design process, and many more.

In this article we will discuss design considerations for the graphical representation of information from the area of Systems Biology given as SBGN (Systems Biology Graphical Notation) maps. This is not a review of the very broad area of combining Bioinformatics and design. Instead, we will focus specifically on the SBGN notation as an example to look into challenges of combining Bioinformatics and design, discuss design decisions for SBGN visualisations based on glyphs and biological network layouts tailored to SBGN maps, and investigate the interactive construction of larger SBGN maps based on small building blocks. Finally, we will present a tool for and some applications with SBGN maps. It should be noted that the authors have been strongly involved in SBGN standard and tool development, have been co-authors of the SBGN standards and most SBGN-related publications, and developed and implemented major parts of the presented software SBGN-ED. This helps to provide deep insights into SBGN design and the design process, and therefore this work is based significantly on previous work of the authors.

The reminder of this article is as follows: in the Introduction, Section 1.1 will briefly introduce the field of systems biology and Section 1.2 will provide an overview about the SBGN standard with the three graphical languages PD, ER, and AF. The part of the SBGN design description will start with glyphs used in SBGN in Section 2.1, then consider how the network based on connected glyphs representing the SBGN map is visualised in Section 2.2, and finally discuss how the network creation and design process could be improved by using predefined building blocks in Section 2.3. The relevant design criteria and decisions are presented in Table 1. Section 2.4 presents SBGN-ED, a tool which implements the described SBGN design, and some applications in biology. The article concludes with Section 3.

**Table 1:** Summary of requirements for the design of glyphs, general map layout, SBGN PD layout, and SBGN Bricks.

Requirements for	Requirement
Design of glyphs	- Glyphs should be simple
	- Glyphs should be scalable
	- Glyphs should be colour-independent
	- Glyphs should be distinguishable
	- Design a minimal number of glyphs
	- Glyphs should have clear semantics
General map layout	- Avoid overlaps between any objects
	- Emphasise map structures
	- Preserve mental map of a user
	- Minimise number of edge crossings
	- Maximise angle between edges
	- Minimise number of edge bends
	- Minimise length of edges
SBGN PD layout	- Vertices must not overlap except in case of containment
	- Draw vertices horizontally or vertically
	- Edges must not overlap border lines of vertices
	- Edges must not overlap each other
	- Draw edges on top of vertices in case of crossings
	- Place a vertex label at least partly inside the vertex
	- A Vertex label must not overlap other vertices or other labels
	- An edge label must not overlap vertices
	- Attach consumption and production edges to opposite sides of process vertices
SBGN bricks	- Provide templates for a wide range of biological patterns
	- Templates should be simple
	- Provide high-quality local layout for the glyphs in a template

### 1.1 Systems biology

**Figure 2:**
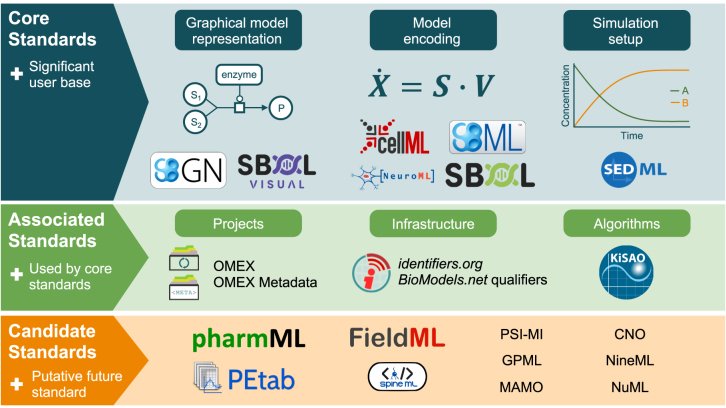
SBGN and other standards covered by COMBINE (from [13]).

The field of systems biology is concerned with the investigation of structure and dynamics of biological processes and functions from a cellular to an organism-wide level, with the aim to understand biological processes on systems-wide levels [14]. A focus is on cellular processes, in particular the analysis of high-throughput data and the modelling of the processes within a cell (sometimes called *systems biology of the cell*). In contrast to some other areas in biology this field has been well formalised, and knowledge has been structured in ontologies and standards for several years. The main body coordinating the development of standards in systems biology is COMBINE, the COmputational Modeling in BIology’ NEtwork [15, 16]. In Figure 2 an overview of COMBINE standards and the position of the Systems Biology Graphical Notation (SBGN) within these standards and associated initiatives is shown. Information about COMBINE and its history can be found, for example, in the work of Myers et al. [17], Hucka et al. [16] and Waltemath et al. [18]. The detailed standards including SBGN specifications have been published as special issues of the *Journal of Integrative Bioinformatics* since 2015 [19].

### 1.2 SBGN

**Figure 3:**
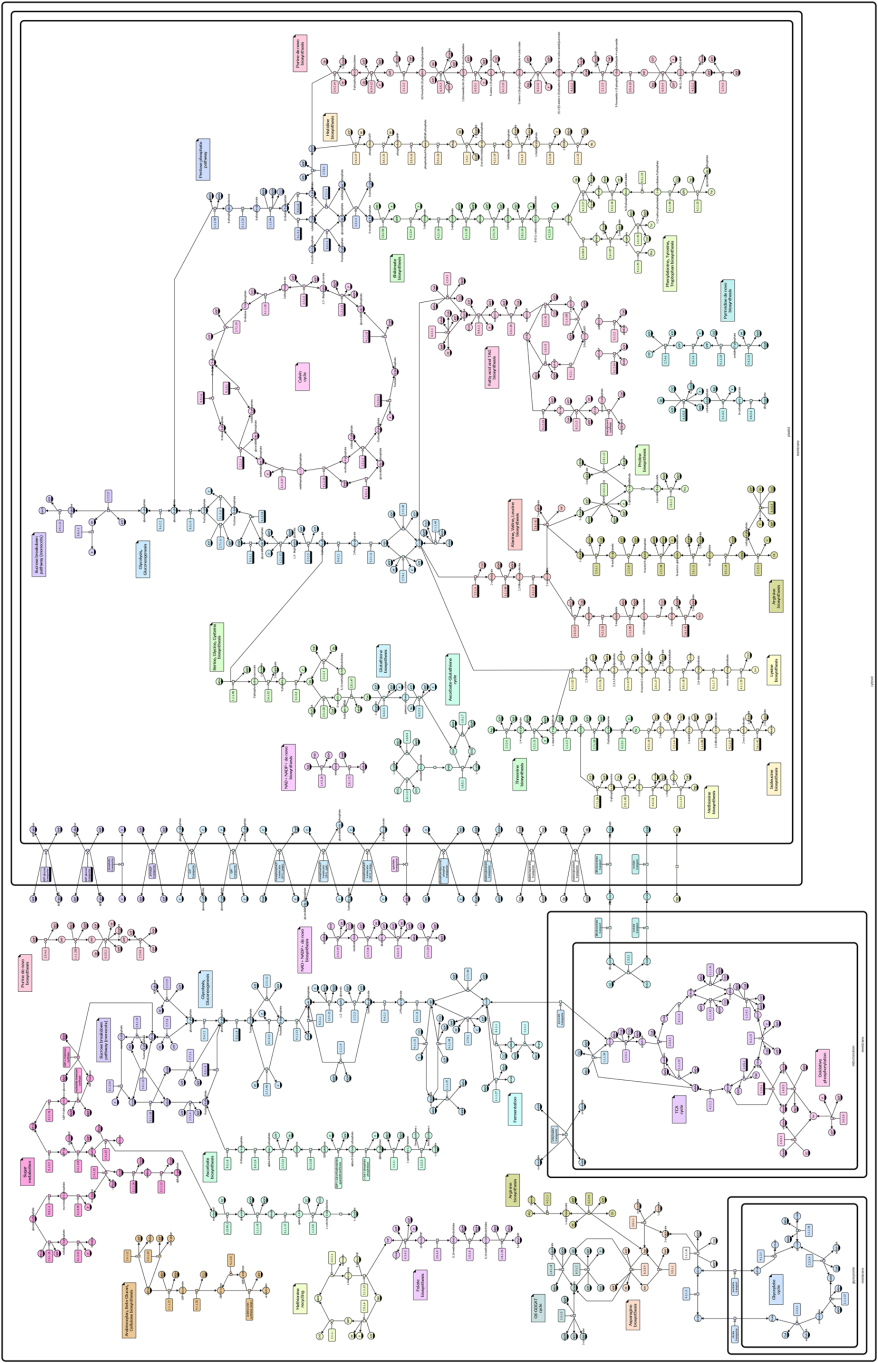
A SBGN map representing major parts of the central metabolism in monocotyledon plants (from [24]).

The Systems Biology Graphical Notation (SBGN) [20] is a formal standard to represent systems biology information visually. The standard is able to include information from cellular processes to organism-wide considerations with a focus on processes from molecular biology, see Figure 3 for an example. SBGN consists of three graphical languages to describe systems biology knowledge from different directions:

– Process Description to show a sequence of interactions between biochemical entities (current standard: SBGN Process Description Level 1 Version 2 [21])
– Entity Relationship to show interactions occurring if the relevant entities are present (current standard: SBGN Entity Relationship Level 1 Version 2.0 [22])
– Activity Flow to show influences between entities (current standard: SBGN Activity Flow Level 1 Version 1.2 [23])

The SBGN map in Figure 3 shows major parts of the network of central metabolism in monocotyledon plants. The information underlying this network has been derived from the MetaCrop database [25, 26] (a system based on Meta-All [27]), a manually curated repository containing information about the metabolism in crop plants such as pathways, reactions, compartments, transport processes, and further details. Individual pathways from MetaCrop have been arranged manually during the design of this map in order to provide a readable and aesthetic visualisation and to fulfil visualisation requirements given in the SBGN PD specification. In addition, colours have been used to add information, here in particular to highlight the different metabolic pathways.

SBGN uses the Systems Biology Ontology [28] (a set of terms commonly used in systems biology), exchange of graphical information is supported by SBGN-ML [29, 30] (an XML-based file format describing the geometry of SBGN maps), and several tools exist to work with SBGN, for example CellDesigner [31], Newt [32], PathVisio [33], SBGN-ED [34], and yEd [35].

## 2 SBGN designs

**Figure 4:**
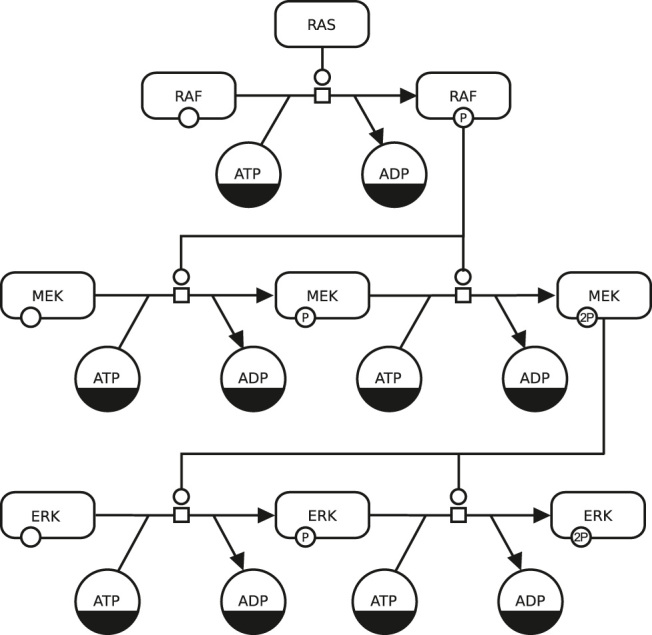
Example of a process description map using two kinds of entity pool nodes: one for pools of different macromolecules and another for pools of simple chemicals. Most macromolecule nodes in this map are adorned with state variables representing phosphorylation states. This map uses one type of process node, the process node, and three kinds of connecting arc, consumption, production, and catalysis. Finally, some entity pool nodes have dark bands along their bottoms; these are clone markers indicating that the same pool nodes appear multiple times in the map (image and text from [21]).

In the reminder of this article, we will focus on one SBGN language, SBGN PD (Process Description, the current standard is SBGN Process Description Level 1 Version 2 [21]). Figure 3 shows biological processes in SBGN PD (short PD in the following), and Figure 4 shows an example from the PD specification. The PD language allows to describe all the processes taking place in a biological system in a direct, sequential, and mechanistic manner.

The language has been designed in a large interdisciplinary team over several years. The design process aimed to cover as much as possible of the known biological processes and mechanisms in a consistent, simple, and precise notation to allow people

– to interpret the PD maps quickly and easily without the need of a legend or other additional descriptions, and
– to exchange the designed graphical descriptions (maps) unambiguously similar to the way engineers would build, use, and exchange electronic circuit diagrams or other formalised drawings.

To achieve this SBGN defines a small but comprehensive set of symbols (glyphs) together with precise semantics and syntactic rules that define the meaning and use of those glyphs. The SBGN PD standard also describes how larger networks should be built and laid out as well as the way in which the graphical information given by the map should be interpreted and used.

### 2.1 SBGN glyphs

*Design a minimal number of simple, easily distinguishable glyphs*.

The term glyph can have several meanings, for example, in architecture it is a vertical mark in the facade of a building, in archaeology a carved or inscribed symbol, and in computer science a (small) graphic symbol that conveys information non-verbally. We use the latter definition of the term.

When designing SBGN PD glyphs a number of requirements were discussed (see also Table 1): Glyphs should be

**Figure 5:**
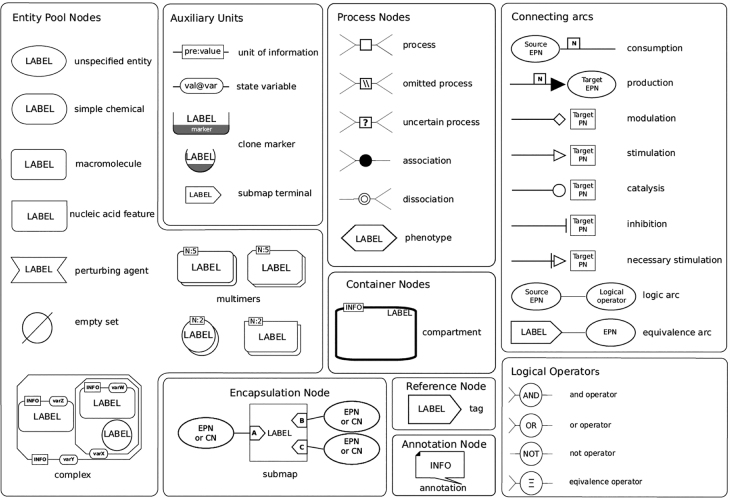
SBGN PD reference card showing all PD glyphs (from [21]).

– scalable, such that maps can be zoomed in and out without losing the shape of the glyphs (e.g., there are no dotted lines in SBGN PD as they would not be well scalable),
– colour-independent, such that a map can also be printed in greyscale, and that colour can be used for additional information not related to SBGN PD (e.g., all glyphs are black/white only),
– simple and only a few, such that glyphs are easy to draw and to memorise, and that it does not require lots of effort to learn SBGN (e.g., the complete number of SBGN PD glyphs is shown in Figure 5), and
– defined with clear semantics, such that the map has a clear meaning and SBGN PD maps could be automatically translated into computable models (e.g., the specification contains formalised and detailed descriptions about meaning and use of SBGN PD glyphs).

Figure 5 shows all glyphs of SBGN PD. The glyphs are structured into groups such as entity pool nodes (vertices), process nodes, connecting arcs and so on, and the design criteria described above are met by those glyphs.

### 2.2 SBGN network layout

*Design an easily readable and meaningful map*.

From a technical point of view a SBGN PD map can be seen as a graph *G* = (*V*, *E*), consisting of a set of vertices *V* and a set of connecting edges *E*, with labels and types attached to vertices and edges. The layout problem of graph *G* thereby refers to the well-studied graph drawing problem. Maps (layouts of graphs) have been produced manually for a long time. Graph drawing algorithms (i.e., automatic layout of networks) are important [36] and have been developed since the last century. Such algorithms compute a map *M* for a given graph/network *G*. The algorithm takes a network or graph and computes a layout (map) *M* consisting of coordinates for the vertices and routings (lines) for the edges.

Examples of such algorithms can be found in the books (e.g., by Di Battista et al. [37] and Kaufmann and Wagner [38]) which also discuss design criteria for good graph layouts. Typical algorithms are force-based methods [39], [40], [41], [42], [43], layered methods [44], [45], [46] and orthogonal or grid-based approaches [47, 48]. Also rules for the creation of biological network maps have been discussed, including choosing layouts, applying colours and using layering and separation [49].

Even though common graph drawing algorithms can be employed for laying out biological networks in general and SBGN maps in particular, domain specific layout methods that conform to representational conventions in biology as well as special approaches have been developed, and there is a large body of literature available, examples include algorithms for specific biological networks such as metabolic networks [9, 50], [51], [52], [53] and protein interaction networks [54], [55], [56], [57], for general biological networks [58], [59], [60], [61], [62], [63], [64], [65], [66], for 2.5D layouts [67, 68] and 3D layouts [69], [70], [71], [72], [73], [74] as well as for specific optimisations or comparisons within the layout [75], [76], [77]. Also open graph drawing problems have been discussed [78].

Criteria for good layouts (i.e., a good design of the map) have been discussed in the before-mentioned books and also in several studies such as [79], [80], [81], [82], [83], [84], [85]). These criteria (see also Table 1) include

– minimising the number of edge crossings,
– maximising the angle between edges,
– minimising the number of edge bends,
– minimising the length of edges,
– avoiding overlaps between objects (vertices, edges, vertices and edges),
– emphasising structures such as clusters, circles and similar, and
– preserving the mental map of a user.

There are additional design requirements for SBGN PD maps, which are given in the SBGN PD specification (see also Table 1). Such design rules include requirements such as

– vertices are in general not allowed to overlap, only when they contain other vertices representing complex molecules or cellular compartments they can be contained within each other (overlap),
– edges must be placed on top of vertices in case the edges cross the vertices,
– there is no overlap allowed between the border lines of vertices and edges and also not between two edges,
– vertices should be drawn horizontally or vertically (but not with an arbitrary angle),
– there are special vertex–edge connections, for a vertex (glyph) representing a process the edges to consumption and production arcs are attached to the centre of opposite sides of the vertex,
– vertex labels have to be placed at least partly inside the vertex and are not allowed to overlap other vertices or other labels, and
– edge labels are not allowed to overlap (cover) vertices

as well as less strict recommendations which should be followed as much as possible and generally improve the clarity of the map.

**Figure 6:**
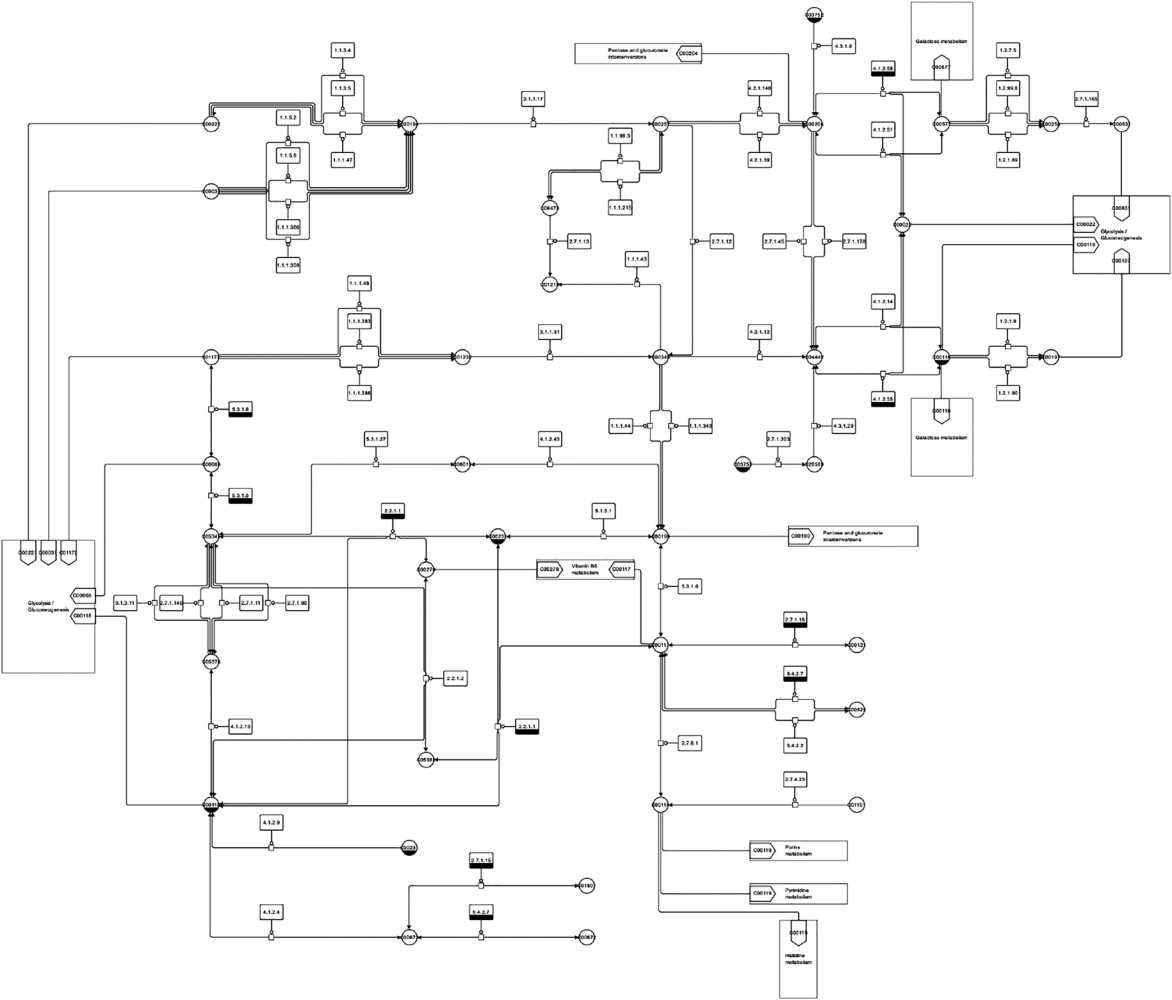
SBGN PD map with automatic layout derived from a KEGG diagram.

Some layout algorithms specific for SBGN PD maps have been developed [86, 87], and Figure 6 shows an example of a computed layout of a SBGN PD map derived from a KEGG pathway diagram which has the advantage that some aspects of the layout, in particular the overall distribution of vertices, is already given by the KEGG diagram [88, 89]. Still, many SBGN maps available are manually drawn as existing layout algorithms still have their limits.

### 2.3 SBGN bricks

*Design a larger map from reoccurring small parts*.

In general, SBGN maps are assembled by drawing SBGN glyphs (see Section 2.1) on a piece of paper or a whiteboard or by placing SBGN glyphs on the canvas of a software which supports SBGN. The glyphs are then connected by SBGN arcs (for examples, see Figure 5, top-right) to create a complete SBGN map. This approach works well for SBGN maps containing 20 to 30 glyphs but turns into a tedious process when designing maps containing several dozens or even hundreds of glyphs.

However, information in systems biology presented in the form of biological networks (SBGN maps) shows different reoccurring patterns which represent different biological concepts. Thus, the SBGN Bricks have been designed which extend the glyph concept of SBGN towards reusable patterns or templates for a wide range of biological patterns. A first version of the SBGN Bricks was introduced by Junker et al. [90] providing an SBGN Bricks dictionary with building blocks of the aforementioned patterns which can be assembled into SBGN maps. A second, more formalised, version has been developed by Rougny et al. [91] structuring the SBGN Bricks into an ontology – the Bricks Ontology (BKO).

**Figure 7:**
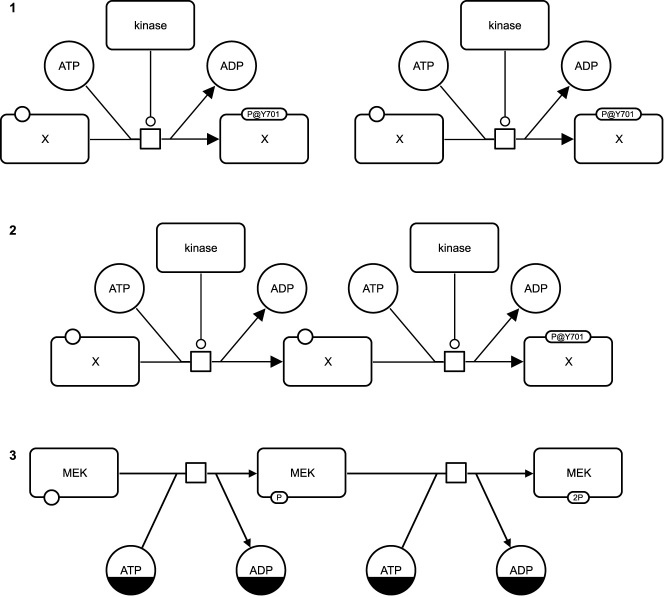
Example for the design of a SBGN PD map using SBGN PD bricks. The two processes in the middle of Figure 4 are assembled using the brick for protein phosphorylation: (1) the template for the protein phosphorylation is placed twice on the canvas, (2) the two glyphs in the centre are merged into one glyph, (3) the labels of the glyphs are changed. Additionally, the layout is adjusted manually. Notes: The glyph “kinase” has been removed from step 3 since it is redundant and is provided by the brick for the first process (not shown here). For a valid SBGN map the glyphs “ATP” and “ADP” require the *clone marker*, an SBGN concept indicating that a glyph appears multiple times on a map.

The SBGN Bricks dictionary provides bricks for patterns in all three SBGN languages covering a wide a range of biological networks and biological concepts, for an example see Figure 7, top. The bricks have been designed manually including all SBGN glyphs and SBGN arcs required to represent a particular biological concept. Bricks are provided as templates with generic glyph labels for integration in software which supports SBGN. SBGN Bricks have two advantages compared to SBGN glyphs when an SBGN map is designed (see also Table 1): they allow for a faster design of, particularly larger, SBGN maps by providing reusable patterns, and they provide a high-quality local layout for the glyphs of each pattern.

Designing an SBGN map using the SBGN Bricks with a software requires three steps: (1) placing the necessary patterns from the SBGN Bricks dictionary on the canvas, (2) merging respective glyphs to connect the patterns into a complete SBGN map, and (3) changing labels of the glyphs accordingly. In an additional, optional step the layout of the resulting SBGN map might require adjustments, either manually or automatically using an algorithm (see also Section 2.2). Figure 7 shows how the two processes in the middle of Figure 4 can be assembled using SBGN Bricks.

### 2.4 SBGN-ED

*Design a tool which implements all design decisions regarding glyphs, layout, and interaction*.

Several software tools have been mentioned above (see Section 1.2) which can be used to work with SBGN. In this section, SBGN-ED [34] will be discussed in more detail.

SBGN-ED has been developed as an add-on for Vanted [92, 93], a framework for the visualisation and analysis of (biological) networks containing experimental data. Thus, SBGN enables users to work with SBGN map and also to use all the functionality Vanted provides. At its core, SBGN-ED allows users

– to create and edit SBGN maps in all three languages using the SBGN glyphs,
– to validate SBGN maps according to the rules in the SBGN specifications,
– to translate diagrams from the KEGG pathway database [88, 89] into SBGN PD (see Figure 6)
– to translate SBML [94] models into SBGN PD,
– to translate SBGN PD maps into SBGN AF maps [95], and
– to export SBGN maps into several image and file formats including SBGN-ML.

In addition to the features listed above, SBGN-ED was extended to provide the SBGN Bricks dictionary [90] allowing the design of SBGN maps using the SBGN Bricks (see Section 2.3). Furthermore, using functionality provided by Vanted, SBGN-ED also supports interaction for SBGN maps (including general interaction with networks [96], the use of different glyphs in navigation [97], or mental-map preserving interaction [98]) SBGN-ED has been used to design SBGN PD maps in general [99] or in dedicated projects such as RIMAS [100] (Regulatory Interaction Maps of Arabidopsis Seed Development), MetaCrop [25, 26] (a curated repository containing information about the metabolism in crop plants), QSDB [101] (Quorum-sensing database) and the COVID-19 Disease Map [102, 103] (a computational knowledge repository of virus–host interaction mechanisms).

In addition, SBGN-ED has also been applied for the visualisation and layout of genome-scale metabolic networks such as the *Pseudomonas aeruginosa* model developed by Zhu et al. [104], Path2Models [105] (a collection of models automatically generated from pathway resources), and LMME [106] (the Large Metabolic Model Explorer).

## 3 Conclusions

Visualisations are very common in systems biology and related areas, and are used to explore, analyse, and communicate information and knowledge. Such visualisations not only need to be accurate but should also be aesthetically pleasing and informative. Here we use the example of the Systems Biology Graphical Notation (SBGN) to investigate design considerations for graphically presenting information from systems biology. We focus on glyphs and the use of glyphs for types of information, the design of a good graph layout for network representation including SBGN-specific requirements, the concept of bricks for visual network creation based on reoccurring patterns, and finally briefly present a tool which implements those design decision to allow users to easily create SBGN PD maps. We use SBGN PD as an example, but similar design criteria have been developed also for SBGN ER and SBGN AF. We believe that this shows how important well-designed representations and interactions are for easy and intuitive visualisation and exploration of biological information, and we hope that in the future design principles, as, for example, discussed in the *Points of View* Nature Methods series [12] will play a more significant role when developing (interactive) visualisations in Bioinformatics.
